# Surveillance of Bovine Tuberculosis and Risk Estimation of a Future Reservoir Formation in Wildlife in Switzerland and Liechtenstein

**DOI:** 10.1371/journal.pone.0054253

**Published:** 2013-01-21

**Authors:** Janne Marie Schöning, Nadine Cerny, Sarah Prohaska, Max M. Wittenbrink, Noel H. Smith, Guido Bloemberg, Mirjam Pewsner, Irene Schiller, Francesco C. Origgi, Marie-Pierre Ryser-Degiorgis

**Affiliations:** 1 Centre for Fish and Wildlife Health (FIWI), Vetsuisse Faculty, University of Bern, Bern, Switzerland; 2 Institute of Veterinary Bacteriology (IVB), Vetsuisse Faculty, University of Zurich, Zurich, Switzerland; 3 Animal Health and Veterinary Laboratories Agency (AHVLA), Weybridge, New Haw, Surrey, United Kingdom; 4 Institute of Medical Microbiology (IMM), Medical Faculty, University of Zurich, Zurich, Switzerland; 5 Federal Veterinary Office, Animal Health Division, Bern, Switzerland; University College Dublin, Ireland

## Abstract

Bovine tuberculosis (bTB) caused by *Mycobacterium bovis* or *M. caprae* has recently (re-) emerged in livestock and wildlife in all countries bordering Switzerland (CH) and the Principality of Liechtenstein (FL). Comprehensive data for Swiss and Liechtenstein wildlife are not available so far, although two native species, wild boar (*Sus scrofa*) and red deer (*Cervus elaphus elaphus*), act as bTB reservoirs elsewhere in continental Europe. Our aims were (1) to assess the occurrence of bTB in these wild ungulates in CH/FL and to reinforce scanning surveillance in all wild mammals; (2) to evaluate the risk of a future bTB reservoir formation in wild boar and red deer in CH/FL. Tissue samples collected from 2009 to 2011 from 434 hunted red deer and wild boar and from eight diseased ungulates with tuberculosis-like lesions were tested by direct real-time PCR and culture to detect mycobacteria of the *Mycobacterium tuberculosis* complex (MTBC). Identification of suspicious colonies was attempted by real-time PCR, genotyping and spoligotyping. Information on risk factors for bTB maintenance within wildlife populations was retrieved from the literature and the situation regarding identified factors was assessed for our study areas. Mycobacteria of the MTBC were detected in six out of 165 wild boar (3.6%; 95% CI: 1.4–7.8) but none of the 269 red deer (0%; 0–1.4). *M. microti* was identified in two MTBC-positive wild boar, while species identification remained unsuccessful in four cases. Main risk factors for bTB maintenance worldwide, including different causes of aggregation often resulting from intensive wildlife management, are largely absent in CH and FL. In conclusion, *M. bovis* and *M. caprae* were not detected but we report for the first time MTBC mycobacteria in Swiss wild boar. Present conditions seem unfavorable for a reservoir emergence, nevertheless increasing population numbers of wild ungulates and offal consumption may represent a risk.

## Introduction

Tuberculosis is a chronic disease caused by bacteria of the *Mycobacterium tuberculosis* complex (MTBC). The MTBC currently comprises *M. bovis* and *M. caprae*, the causal agents of bovine tuberculosis (bTB), *M. microti*, infecting mainly small wild mammals, *M. pinnipedii,* causing tuberculosis in marine mammals, *M. mungi*, recently described in mongooses, and the primarily human pathogens *M. tuberculosis*, *M. africanum* and *M. canettii* [1]–[4].

Bovine tuberculosis is a disease of global importance. Infection of livestock with *M. bovis* and, to a lesser extent, with *M. caprae*, constitutes a considerable obstacle to international cattle trade [5]. *M. bovis* infections are also of concern for the conservation of endangered species [6]–[8]. Furthermore, both *M. bovis* and *M. caprae* have a zoonotic potential. In the European Union (EU), *M. bovis* accounted for 133 cases of human tuberculosis in 2009, with a case fatality rate of 5%, and sporadic cases of *M. caprae* infection in humans have been reported [9]–[13].

Eradication of bTB in cattle is hampered by the chronic nature of the disease and difficult in vivo testing [14], [15]. Furthermore, the presence of a wildlife reservoir often impedes efforts towards disease control. In Europe, this has been well documented in the United Kingdom (UK), the Republic of Ireland (RoI) and Spain. These countries display the highest bTB prevalences in cattle in the EU and face difficulties controlling wildlife reservoirs in the badger (*Meles meles*) and wild ungulates, respectively [10], [16].

A reservoir consists of a host population (or several epidemiologically linked populations), within which a pathogen persists without the necessity of other species acting as external sources of infection (except for the initial introduction of the pathogen) [17]–[19]. One or several host species in which the pathogen is self-maintained in such a way are called “maintenance host(s)” (or formerly “reservoir hosts”) [20], while the term “spillover host” refers to a species susceptible to infection but in which population the infection is not self-maintained [21], [22]. However, host status may change from “spillover” at low densities to “maintenance” at high densities, when intraspecific disease transmission is facilitated [22].

Among the best-known wildlife reservoirs for tuberculosis worldwide are the brushtail possum (*Trichosurus vulpecula*) in New Zealand [22], [23], the African buffalo (*Syncerus caffer*) in South Africa [24], the white-tailed deer (*Odocoileus virginianus*) in the USA [25], and the bison (*Bison bison*) and elk (*Cervus elaphus manitobensis*) in Canada [26]. In Europe, the badger constitutes the major reservoir in the UK and in the RoI [27], while only single cases or markedly lower prevalences have been reported in this species in continental Europe so far [28], [29]. The wild boar (*Sus scrofa*), red deer (*Cervus elaphus elaphus*) and fallow deer (*Dama dama*) have a reservoir status on the Iberian peninsula [16].

In recent years, bTB has appeared as a (re-)emerging disease in European wildlife, especially in wild ungulates (e.g. [30], [31]). All countries surrounding Switzerland (CH) and Liechtenstein (FL) have been affected, with positive cases found partly in close proximity to the CH and FL borders: *M. caprae* was recently detected in three out of 332 red deer in Southern Germany [32] and infection hot spots have developed in western Austria, where bTB prevalence in red deer locally exceeds 40% [33]. In Northern Italy, *M. bovis* has been previously diagnosed in 3% of wild boar [34], and few cases were recently detected in close proximity to the Swiss border (M. Pacciarini, personal communication).

In Switzerland (CH), the last documented cases of bTB in wildlife date back to the 1950′s, before the country officially gained TB-free status in 1960, and involved badgers, roe deer (*Capreolus capreolus capreolus*), Alpine chamois (*Rupicapra rupicapra rupicapra*) and red deer [35], [36]. A cross-sectional study carried out in 2002/2003 in 69 wild boar from the canton of Ticino (southern CH), suggested that *M. bovis* was absent in this region [37]. Three animals showed lymph node lesions suggestive of bTB but *M. avium-intracellulare* was isolated in all cases. However, the validity of this study was limited, as attempts to culture live organisms were restricted to these three samples that had macroscopic bTB-like lesions. In another study, attempts to culture *M. bovis* or *M. tuberculosis* from tissue pools of more than 320 farmed cervids from CH were unsuccessful [38].

Considering the recent emergence of bTB in neighboring countries, our goal was to assess the current situation in CH and FL wildlife and to provide baseline data for future investigations. We conducted a cross-sectional prevalence study in geographical areas considered at highest risk, using as target species the potential reservoir hosts wild boar and red deer. Furthermore, we reinforced the existing national scanning surveillance programs in all wild mammals regarding bTB. Finally, we reviewed risk factors associated with the maintenance of bTB in wildlife, as identified in countries with a recognized reservoir, and assessed their occurrence in CH and FL. We used this information to estimate the probability of the formation of a future *M. bovis*/*M. caprae* reservoir in local wild ungulates in these countries.

## Materials and Methods

Sampling was performed in CH and in FL from the end of September 2009 to the end of February 2011, including two consecutive hunting seasons.

### Ethics statement

This study did not involve purposeful killing of animals. All samples originated from dead wildlife legally hunted during hunting season or legally shot because of severe debilitation. According to CH and FL legislation (922.0 hunting law and 455 animal protection law, including legislation on animal experimentation; www.admin.ch and www.gesetze.li), no ethical approval or permit for animal experimentation was required.

### Cross-sectional study

**Figure 1:**
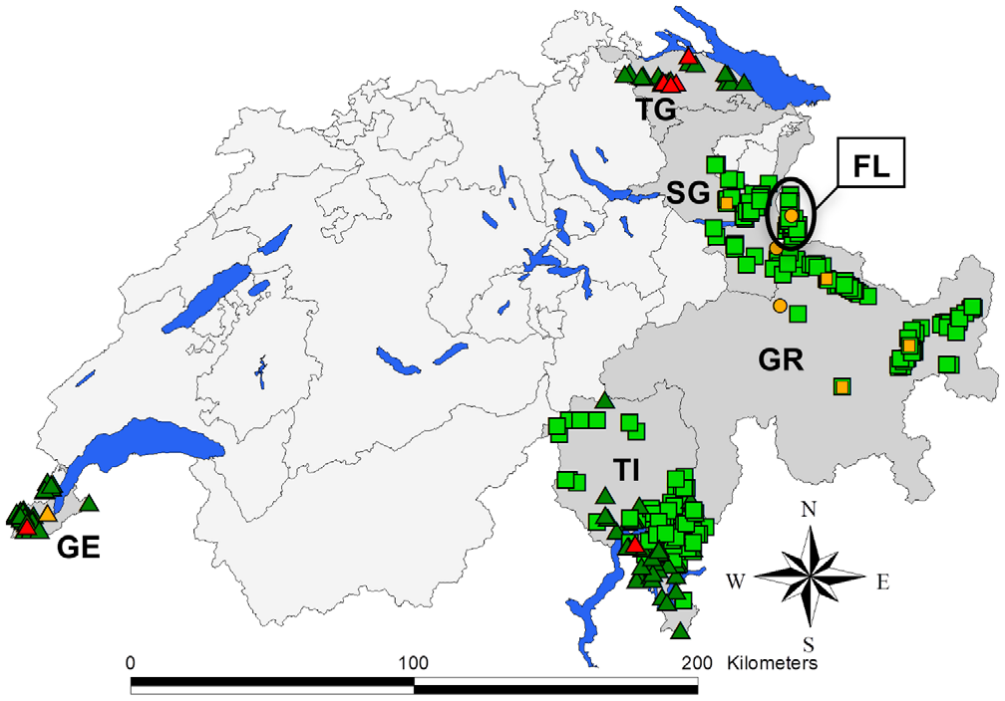
Map of Switzerland and Liechtenstein depicting the origin of samples and microbiological results. Animal species: red deer (square); wild boar (triangle); other species (circle). Microbiological results: survey samples negative for mycobacteria of the *M. tuberculosis*-complex (MTBC; green); MTBC-positive survey samples (red); scanning surveillance samples (all MTBC negative; orange). Study areas (dark grey): Geneva (GE); Thurgovia (TG); Saint Gall (SG); Principality of Liechtenstein (FL); Grisons (GR); Tessin (TI). Further: cantonal borders (grey lines); main lakes (blue areas).

Study sites were selected based on the occurrence of wild boar and red deer, size of the hunting bags (Swiss hunting statistics: http://www.wild.uzh.ch/jagdst/) and on the geographic proximity to neighboring countries where bTB had recently been reported in wildlife. CH is organized in political subunits (cantons) with different hunting regimes. The survey was carried out in the cantons of Geneva, Thurgovia, Saint Gall, Grisons and Tessin, and in FL (Table 1, Figure 1). In Geneva, hunting is prohibited, but the wild boar population is regulated by cantonal game wardens. In Thurgovia and FL, hunters hunt on leased hunting grounds and there are no game wardens. The situation in Saint Gall is the same as in Thurgovia except that cantonal game wardens are present. In Grisons and Tessin, hunters buy licenses allowing them to harvest a certain number of animals per season; they may hunt in any area within the canton during a limited time period and hunting activities are supervised by game wardens.

**Table 1:** Study areas, hunting bags for wild boar and red deer, and red deer population size estimation.

			Wild boar	Red deer	
Study area	Total area	Forest area	Hunting bag 2010	Hunting bag 2010	Population size
	(km^2^)	(km^2^)			
**Liechtenstein**	150	66	0	215	400–500
**Switzerland (total)**	41285	12716	6878	9016	28483
**Geneva**	282	39	491	0	30
**Thurgovia**	991	213	518	4	15
**Grisons**	7105	1897	10	4118	13500
**Tessin**	2813	1373	1019	1776	4900
**Saint Gall**	2025	620	68	577	1025

Population estimates for red deer were assessed by head light counts and observations of game wardens and hunters. Population estimates for wild boar are only locally available (see Table 4 for density data). Sources: Statistic Yearbook Liechtenstein 2011 (Statistical Office Liechtenstein); Swiss hunting statistics (Federal Office for the Environment, FOEN); Swiss Statistics 2011 (Federal Statistical Office, FSO).

Tissue specimens were collected from a convenience sample of red deer and wild boar from the regular hunting bag. In the cantons of Geneva and Tessin, sampling was performed by staff of the Centre for Fish and Wildlife Health (FIWI). In the other areas, game wardens and hunters were asked to collect tissues and submit them to the FIWI immediately after collection. They were previously instructed for tissue identification and sample collection via lectures and demonstrations on carcasses. Tissue collection was carried out from the carcasses and organs after evisceration. Collected tissues per animal comprised the mandibular lymph nodes (ML), medial retropharyngeal lymph nodes (RL), palatine tonsils (PT), mediastinal lymph nodes (MedL), and the mesenteric lymph nodes (MesL). Sampling sets consisted of detailed sampling instructions, pre-labeled bags for each tissue, a pair of latex gloves, and a data sheet to gather information on species, sex, estimated age, body condition, date of death and location, and the presence of macroscopic lesions on the carcass. According to the age estimated by the submitter, animals were grouped into three categories: Juvenile (less than one year old), subadult (one to two years old) or adult (over two years old). Adequate packaging for mail delivery was provided, and shipping costs were covered by the project and the cantonal hunting offices.

Calculation of sample size per species and sampling area was based on estimated population sizes derived from the regional hunting bags, and performed using WinEpiscope® 2.0 software [39], with the aim of detecting infection and assuming a prevalence of 5% in each species with 95% confidence level. Target values for wild boar were 59 animals from Geneva, 58 from Thurgovia, and 58 from Tessin. Target values for red deer were 59 animals from Saint Gall, 58 from Grisons, 58 from Tessin, and 56 from FL.

In total, 434 free-ranging ungulates (165 wild boar and 269 red deer) were sampled and the targeted sample size was met or nearly met in all study areas (Table 2). All required tissues were obtained from 36% of the sampled animals (n = 158), while one or more lymph nodes were not available for the others (n = 277).

**Table 2:** Sex and age distribution per study area of hunted wild boar and red deer sampled from 2009–2011 in Switzerland and in Liechtenstein.

		Young			Yearling			Adult			No data			Total
Species	Area	F	M	Nd	F	M	Nd	F	M	Nd	F	M	Nd	
**Wild boar**	**Total**	**24**	**24**	**1**	**30**	**28**	**1**	**25**	**25**	**2**		**3**	**2**	**165**
	GE	15	8		14	11		8	5					61
	TG	4	9		6	3		5	3					30*
	TI	5	7	1	10	14	1	12	17	2		3	2	74
**Red deer**	**Total**	**35**	**29**		**36**	**26**		**65**	**73**		**1**		**4**	**269**
	FL	10	7		4	9		9	7		1		1	48
	GR	9	10		6	8		31	19				2	85
	SG	12	6		5	6		11	7					47
	TI	4	6		21	3		14	40				1	89

* Due to organizational reasons, sampling took place only during the 2010/11 hunting season. Study areas: Geneva (GE); Thurgovia (TG); Tessin (TI); Liechtenstein (FL); Grisons (GR); Saint Gall (SG). Sex: female (F); male (M); no data (Nd).

### National scanning surveillance programs

Within the existing national surveillance programs for wildlife health in CH and FL, game wardens and hunters are regularly encouraged to submit animals presenting disease signs or found dead to the FIWI for a post-mortem analysis free of charge. During the present project, the awareness of hunting authorities, game-wardens and hunters for bTB as a currently emerging disease was increased by articles published in hunting magazines and oral communications in the frame of courses and of the information campaign surrounding the cross-sectional study. Field partners were asked to systematically submit carcasses, organs or samples of all wild mammals presenting lesions suggestive of tuberculosis, independently of the species and geographic region.

Samples or carcasses of eight diseased animals (one wild boar, four red deer, one roe deer, one Alpine chamois and one Alpine ibex *Capra ibex ibex*) were included in the project: five animals were seen as potentially tuberculous by the submitter, and three were sent for bTB-unrelated routine diagnostics but presented tuberculosis-like lesions (TBL, see “Macroscopic evaluation and pooling” for definition) at necropsy and were therefore sampled for further investigation. Tissue selection and collection for microbiological analyses were carried out according to the same protocol as for hunted, apparently healthy animals, except that organs with TBL were additionally collected. Organs presenting lesions were also systematically sampled for histology to determine the cause of disease. These samples were fixed in 10% buffered formalin and embedded in paraffin. Five-micron-thick tissue sections were obtained and stained with hematoxylin and eosin. Special stainings, including Ziehl-Neelsen, Gram, Grocott, and immunohistochemistry were applied to selected samples as needed. General bacteriological cultures were performed accordingly on selected samples following accredited protocols (Institute of Veterinary Bacteriology, University of Bern).

### Macroscopic evaluation and pooling

Tissue material from all sampled animals (cross-sectional study and scanning surveillance) was evaluated macroscopically by qualified staff, either on-site if FIWI staff conducted sampling in the fields, or in the necropsy hall if samples were shipped by mail. Common lesions in tuberculous wild ungulates consist of caseo-granulomas of various sizes which can frequently become mineralized (e.g., [40], [41]), or of purulent lesions, notably in Cervids [42]. Sampled tissues were classified as presenting TBL if white-tan caseous-necrotic to purulent lesions of any size and consistency with or without mineralization were detected.

Half of each lymph node and tonsil was pooled per animal for microbiological analysis. Between animals, instruments were thoroughly decontaminated with 5% Amocid® (Lysoform, Berlin, Germany) and cutting board cover was changed. Subsequently, all samples were stored at −20°C and pools were sent frozen to the Swiss National Center for Mycobacteria at the Institute of Veterinary Bacteriology, University of Zurich, where microbiological analysis was performed.

### Microbiological analysis

#### Culture

All tissue handling was conducted in a laminar flow cabinet. A sterile set of surgical instruments and a new cutting surface were used for each pool. Of each tissue pool, about 2 g of material were taken for analysis, including gross lesions, if present. The tissue was mixed with 14 ml saline solution (0.9%), homogenized with an ULTRA-TURRAX® Tube Drive Workstation (IKA®, Staufen, Germany) and filtered through sterile gaze. Of this suspension, 1.5 ml were centrifuged for 20 minutes at 16000×*g* and the pellet was frozen and preserved for PCR analysis. The remaining suspension was decontaminated for 15 minutes with 4 ml H_2_SO_4_ (4%) at room temperature, neutralized with 5.6 ml NaOH (1N) and buffered with 20 ml sterile phosphate buffered saline (PBS), according to document WHO/TB/98.258: Laboratory Services in Tuberculosis Control, Part III, Culture, pp. 37–42. After centrifugation for 20 minutes at 4000×*g* and 15°C, the supernatant was discharged and the pellet resuspended with 2 ml sterile PBS. Of this basal suspension, 0.5 ml were added into a vial of liquid medium BD BACTEC^TM^ MGIT^TM^ Tube (7 ml; Becton, Dickinson and Company, Franklin Lakes, New Jersey, United States) containing modified Middlebrook 7H9 broth base and an integrated fluorescent indicator. This mixture was enriched with 0.8 ml BD BACTEC^TM^ MGIT^TM^ 960-Supplement (Becton, Dickinson and Company, Franklin Lakes, New Jersey, United States) containing PANTA (Polymyxin B, Amphotericin B, Nalidixic acid, Trimethoprim, Azlocillin) antibiotic mixture and growth supplement, and incubated in a BACTEC^TM^ MGIT^TM^ 320 incubator (Becton, Dickinson and Company, Franklin Lakes, New Jersey, United States) at 37°C for eight weeks.

Of the same basal suspension, 0.2 ml were inoculated on each Middlebrook 7H11 Medium slant agar (Becton, Dickinson and Company, Franklin Lakes, New Jersey, United States) and Löwenstein-Jensen Medium slant agar (Becton, Dickinson and Company, Franklin Lakes, New Jersey, United States), supplemented with Glycerin and PACT (Polymixin B, Amphotericin B, Carbenicillin, Trimethoprim). Solid media were incubated at 37°C for at least eight weeks and checked regularly for growth. Cultures were considered positive if typical growth occurred and acid-fast bacilli were detected with Ziehl-Neelsen staining. In this case, DNA was extracted as described below. Media that showed no growth after twelve weeks were considered negative. All incubation procedures were conducted in a biosafety level 3 (BSL3) laboratory.

To verify the obtained results, analysis was repeated on all wild boar samples showing TBL (n = 17). Two modifications to the above mentioned protocol were made to enhance the chance for cultivation of slow-growing mycobacteria of the MTBC: Tissue material was decontaminated using N-Acetyl-L-*Cystein-*NaOH (NALC-NaOH) from the BD MycoPrep^TM^ Specimen Digestion/Decontamination Kit (Becton, Dickinson and Company, Franklin Lakes, New Jersey, United States) instead of H_2_SO_4_ and NaOH [43] and the incubation time of liquid media was prolonged to twelve weeks.

#### DNA extraction

DNA extraction from frozen tissue pellets was performed using the MagNA Pure LC DNA isolation kit II for mammalian tissue and the automated MagNA Pure LC instrument (both: Roche Diagnostics, Basel, Switzerland) according to the manufacturer's protocol, with an external Proteinase K digestion step. For mechanical disruption, 200 µl Tissue Lysis Buffer (Roche Diagnostics, Basel, Switzerland) was added to the pellet, and samples were homogenized twice using tubes containing ceramic beads (Omni International, Kennesaw, United States) and a Precellys 24 homogenizer (Bertin Technologies, Montigny, France) for 45 sec. at 6.500 rpm. The samples were centrifuged for 2 min at 13.000 rpm and 80 µl of the supernatant were added to 20 µl Proteinase K (Roche Diagnostics, Basel, Switzerland) and incubated at 60°C for 30 minutes. After digestion, samples were centrifuged again for 1 minute at 8000 rpm and the supernatant was transferred to the sample cartridges. Setting of the MagNA Pure LC instrument was done according to the manufacturer's protocol.

For DNA isolation from cultured bacteria, either one loop of colony material from slant agar suspended in 400 μl NaCl (0.9%), or 400 μl of liquid culture, were inactivated at 95°C for 30 minutes using a BioShake IQ (analytik Jena, Jena, Germany). Bacteria were lysed by addition of 50 μl lysozyme (10 mg/ml) and an overnight incubation step at 37°C shaking at 900 rpm, followed by mechanical disruption as described above. DNA was extracted using the QIAGEN DNeasy blood and tissue kit (Qiagen GmbH, Hilden, Germany) in accordance with the manufacturer's protocol.

#### DNA amplification and molecular testing

DNA extracts were analyzed at the Institute of Medical Microbiology, Zurich. PCR analysis for detection of MTBC was done with the COBAS® TaqMan® MTB Test kit (Roche Diagnostics, Basel, Switzerland) according to the manufacturer's instructions. Detection of MTBC DNA is based on primers amplifying a conserved region of the 16S ribosomal RNA gene in combination with a MTBC specific Taqman probe [44].

PCR amplification was carried out using the COBAS® TaqMan® 48 Analyzer (Roche Diagnostics, Basel, Switzerland) in 100 μl-reaction mixtures containing 50 μl of freshly made primer-master mix solution (Roche Diagnostics, Basel, Switzerland) and 50 μl of extracted DNA solution. If PCR inhibition was observed (wild boar samples number TI132, TI133, TI134, TI135, TG413 and TG435; presumably due to tissue contaminants), the DNA samples were 5x diluted in lysis/elution buffer (v:v, 1:1) of the Roche respiratory DNA extraction kit (Roche Diagnostics, Basel, Switzerland) and reanalyzed using the COBAS® TaqMan® MTB Test kit. Negative and positive control reactions were performed with material supplied in the test kit.

If culture yielded acid-fast bacilli that were negative by PCR on grown colonies for MTBC DNA, these were classified as atypical mycobacteria and not further differentiated.

#### Genotyping

If DNA specific for MTBC mycobacteria was successfully amplified from cultured material, genotyping was performed using the GenoType® MTBC kit (HainLifescience GmbH, Nehren, Germany) according to the manufacturer's protocol. Briefly, PCR amplification was carried out in reaction mixtures containing 35 μl of primer-nucleotide-mix (Hain Lifescience GmbH, Nehren, Germany), 5 μl 10x PCR buffer for HotStarTaq (Qiagen GmbH, Hilden, Germany), 2 μl 25 mM MgCl2 solution (Qiagen GmbH, Hilden, Germany), 0.2 μl HotStarTaq (Qiagen GmbH, Hilden, Germany), 3 μl H_2_O and 5 μl of DNA positively tested in the PCR assay. Reverse hybridization of the amplified products was performed and the test strips were interpreted, both in accordance with the protocol provided by the manufacturer.

#### Spoligotyping

Six DNA samples originating from tissue pellets were spoligotyped at the Animal Health and Veterinary Laboratories Agency, Weybridge, UK, according to the method of Kamerbeek et al. [45] with minor modifications according to Cadmus et al. [46] and then assigned International Spoligotype names by www.Mbovis.org [47].

#### Statistical analysis

The two-tailed Fisher's Exact Test was used to compare the occurrence of TBL and atypical mycobacteria between wild boar and red deer, as well as among sexes, age categories and sampling areas within each species. Significance level for each test was set at <0.05. Statistical analysis, including the calculation of 95%-confidence intervals for bTB and MTBC prevalence, was performed using NCSS 2007 statistical software (Version 07.1.15; Kaysville, UT, USA).

### Risk factor assessment

#### Literature review

Four online databases (PubMed, ISI Web of Knowledge, EBSCOhost and Google Scholar) were first searched for information on wildlife maintenance hosts for bTB worldwide, using the key words “bovine tuberculosis”, “wildlife” and “reservoir”. Scientific articles considered relevant according to the abstract were selected for detailed reading, and constituted the basis for further targeted search for articles documenting wildlife bTB reservoirs worldwide and risk factors favoring the maintenance of bTB in these host species. We defined a specific factor as a “risk factor” in this review, (1) if it had been shown to be associated with the prevalence or presence of bTB or TBL in the respective reservoir host (excluding individual factors such as sex and age), (2) if such a role was suggested by the author(s), or (3) if a factor was present that had been suggested or shown to play an important role in bTB maintenance in another country (e.g. intensive wildlife management shown as risk factor in Spain, present also in Portugal).

Furthermore, we attempted to compile comparable data on bTB prevalence and population density of reservoir species and selected spillover hosts. Comparability required (1) availability of prevalence and density data from the same geographical area, and (2) use of the same methods for prevalence and density estimations, respectively, in the different regions. Since culture is considered the gold standard for mycobacterial diagnostics [48], these data were preferred for prevalence estimation. If not available, prevalence estimates based on other diagnostic tests were considered.

#### Telephone survey

The situation regarding risk factors identified in the literature review was assessed for CH and FL. We reviewed the current legislation and conducted a telephone survey with officials of the hunting administrations of the study areas.

## Results

### Cross-sectional study

**Figure 2:**
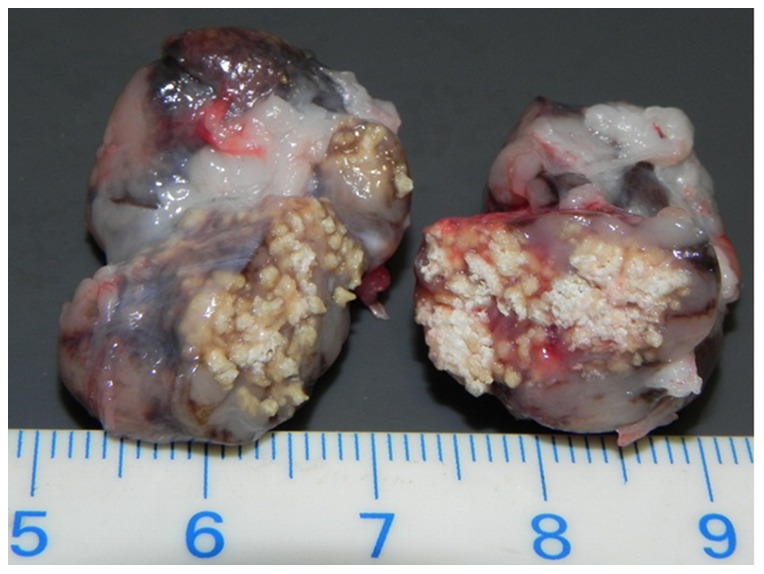
Bilateral tuberculosis-like lesions in the mandibular lymph nodes of a wild boar. This sample was positive for mycobacteria of the *Mycobacterium tuberculosis* complex on tissue material, but yielded only atypical mycobacteria in culture. Scale: centimeters.

We detected TBL in 17 wild boar (10.3%) and five red deer (1.9%). This difference between species was significant (p = 0.0002). We did not observe any case of generalized lesions in either species. In the majority of wild boar (n = 11), lesions consisted of focal to multifocal white-yellow calcified to caseo-calcified foci of 0.1–1.5 cm in diameter (Figure 2), frequently surrounded by a fibrotic capsule, but we also observed firm light yellow nodules (0.2–1.5 cm in diameter) that were concentrically layered and surrounded by a fibrotic capsule (n = 4). Two further individuals had both lesion types in different tissues. Lesions were generally confined to a single anatomical site and restricted to tissues of the head, but two wild boar each presented changes in two and three different sites, respectively, and a single animal showed lesions in the MesL. Wild boar from Thurgovia and Geneva showed significantly more frequently TBL than wild boar from Tessin (p = 0.0073 and p = 0.0012, respectively), while differences among sexes and age categories were not significant.

Lesions in red deer consisted of purulent tonsillitis (n = 2) or miliary calcified lesions (n = 3), all confined to a single anatomical site. Differences among sexes, age categories and sampling areas were not significant.

**Figure 3:**
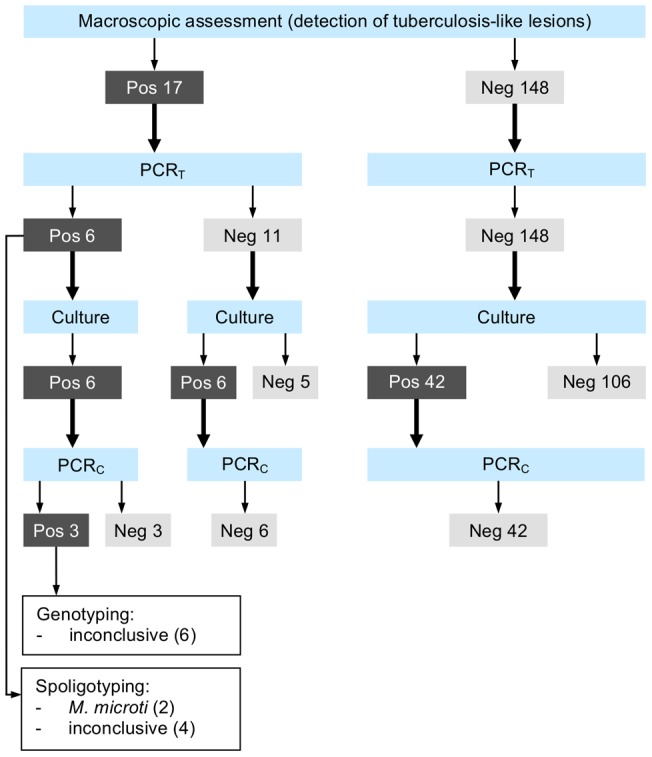
Macroscopic and microbiological results, wild boar. Real-time PCR from tissue material (PCR_T_) and real-time PCR from culture (PCR_C_) for the detection of mycobacteria of the *Mycobacterium tuberculosis*-complex (MTBC). Positive mycobacterial cultures negative by PCR_C_ were classified as atypical mycobacteria. Cultures were considered positive if typical growth occurred and acid-fast bacilli were subsequently detected with Ziehl-Neelsen staining.

**Figure 4:**
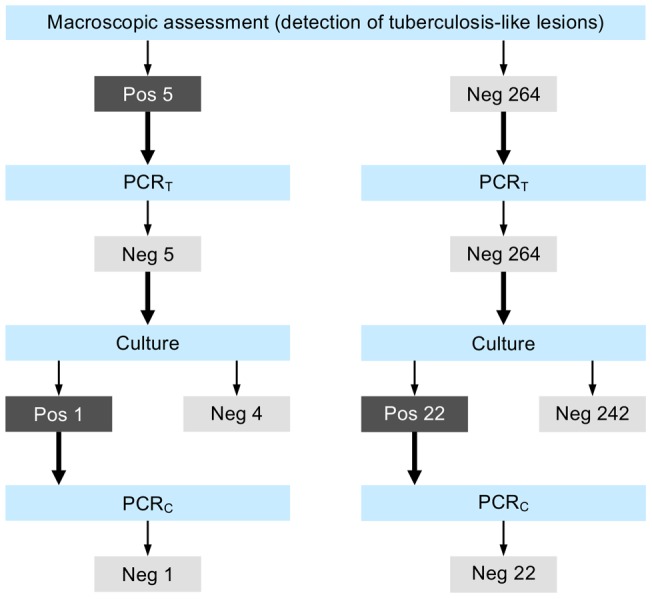
Macroscopic and microbiological results, red deer. Real-time PCR from tissue material (PCR_T_) and real-time PCR from culture (PCR_C_) for the detection of mycobacteria of the Mycobacterium tuberculosis-complex (MTBC). Positive mycobacterial cultures negative by PCR_C_ were classified as atypical mycobacteria. Cultures were considered positive if typical growth occurred and acid-fast bacilli were subsequently detected with Ziehl-Neelsen staining.

Detailed laboratory results for wild boar and red deer are presented in Figures 3 and 4. In wild boar, mycobacteria of the MTBC were detected in six animals (GE403, TG422, TG435, TG454, TG481 and TI135) by direct real-time PCR on tissue samples (PCR_T_; apparent MTBC prevalence: 3.6%, 95% CI 1.4–7.8%), all of which presented TBL (Figure 1). Spoligotyping lead to the identification of *M. microti* in two cases (TG435 and TG481), while an inconclusive banding pattern was obtained in the other four cases (TG422, TG454, GE403 and TI135). Presence of MTBC mycobacteria was subsequently also detected by culture and PCR_C_ for the two *M. microti*-positive animals and for TG422, but genotyping yielded inconclusive banding patterns. Culture material from TG422 was only weakly and transiently positive for MTBC mycobacteria, and thus no appropriate material could be obtained for further spoligotyping.

Atypical mycobacteria were cultured from 54 wild boar (32.7%), including all six MTBC-positive animals and six further animals with TBL. Culture growth was significantly more often observed in samples from wild boar with TBL than from individuals without visible lesions (p = 0.0009), while differences among sexes, age categories and sampling areas were not significant.

In red deer, all samples were tested negative for mycobacteria of the MTBC both by PCR_T_ and PCR_C_ (apparent MTBC prevalence: 0.0%, 95% CI 0–1.4). Atypical mycobacteria were cultured from 23 red deer (8.6%), including one animal with TBL. Overall, culture growth was more often observed in adults than in juveniles and subadults (p = 0.0479), but this difference was not significant when study areas were considered separately. Differences among sexes and sampling areas were not significant either. Atypical mycobacteria were significantly more often detected in wild boar than in red deer (p = 0.0000).

Overall, *M. bovis* and *M. caprae* were not found in the investigated samples, but detected MTBC mycobacteria could not be identified to species level in four wild boar.

### Scanning surveillance

Combined macroscopic, histologic and bacteriologic examinations showed that the four red deer presented, respectively: a purulent lymphadenitis of a cervical lymph node; a severe bronchopneumonia; a lymphadenomegaly in association with myositis; and multifocal parasitic granulomas in the mesentery. The wild boar displayed multifocal mineralized foci within the liver parenchyma of presumptive parasitic origin. The chamois showed a granulomatous peritonitis and hepatitis that were due to presumptive parasitic infestation as well. In the ibex, the enlargement of mesenteric and pulmonary lymph nodes mentioned by the submitter was not confirmed at necropsy; this animal only presented a mild lymphoid hyperplasia of the pulmonary and mesenteric lymph nodes. The roe deer was diagnosed with a multisystemic lymphosarcoma.

All eight cases were tested negative for mycobacteria both by PCR_T_ and by mycobacterial culture.

### Risk factor assessment

#### Literature review

**Figure 5:**
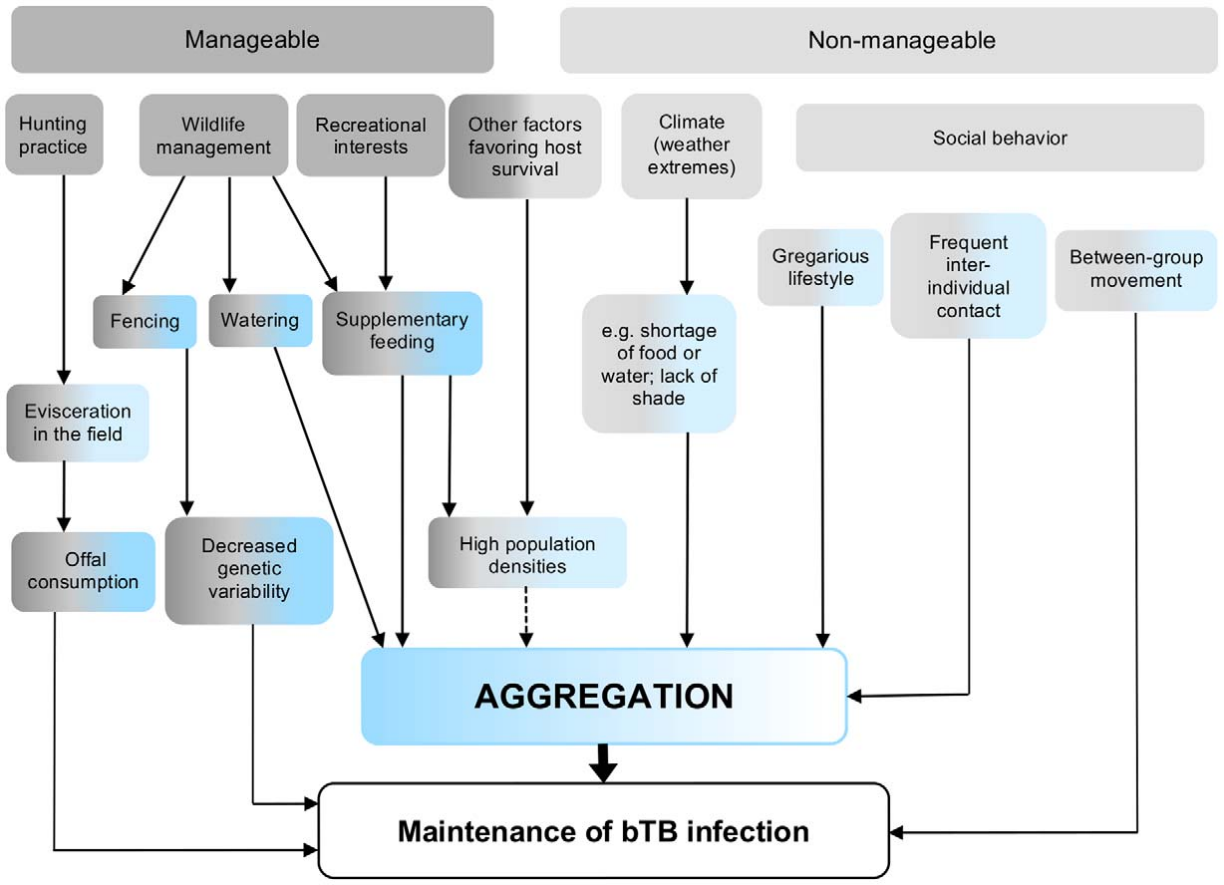
Risk factors favoring the maintenance of bovine tuberculosis (bTB) in reservoir host populations.

Results of the literature review are summarized in Figure 5 and Table 3. The main recognized risk factor for bTB maintenance worldwide appears to be the aggregation of animals. “Aggregation” needs to be distinguished from “density” (the number of individuals per surface unit). Here, we define aggregation as a gathering of individuals in any localized area. The distance between individuals is short up to physical contact, and congregation is usually triggered by a central point of attraction.

**Table 3:** Population densities and prevalences of bovine tuberculosis (bTB) in documented wildlife maintenance and spillover hosts.

	Country	Host(s)	BTB Prevalence		Density		References
					(individuals/km^2^)		
		Maintenance	Reported	Category	Reported	Category	
**1**	Spain	Wild boar	47%	High	1 – 90	High	[16], [53], [101]
		Red deer	12.35 %	Medium	30.9	High	[16], [102]
**2**	Portugal	Wild boar	15.9%*	Medium	No data		[30], [55]
		Red deer	10.3%*	Medium	No data		[16], [55]
**3**	Great Britain	Badger	<17.7%	Medium	25.3	High	[103]–[105]
**4**	Republic of	Badger	14.1%	Medium	No data^a)^	-	[27], [106]
	Ireland						
**5**	USA (Michigan)	White-tailed deer	3.5%	Low	19–23	High	[25], [42]
**6**	Canada	Elk	1%	Low	0.15–0.25	Low	[26], [59], [107]
		Wood bison	49%	High	0.011 -	Low	[26], [56]
					0.162		
**7**	New Zealand	Brushtail possum	up to 10%^c)^	Low-	Frequently	High	[22], [23]
				medium	> 300^b)^		
**8**	South Africa	African buffalo	47%	High	0.9–1.4	Low	[24], [108]
		**Spillover**					
**9**	Germany	Red deer	0.91%	Low	1.5–7 ^c)^	Low-	[32]
						medium	
**10**	Italy	Wild boar	3%	Low	1.7	Low	[34], [109], [110]
**11**	USA	White-tailed deer	0.4 – 1.2% ^d)^	Low	1.8–2.5^ d)^	Low	[100]
	(Minnesota)						

* Culture performed only on animals presenting bTB-compatible lesions; a) total number of badgers in the Republic of Ireland (approximately): 84000 [111], b) general data for uncontrolled possum populations; local densities as high as 1000 ind./km^2^ [22] and local TBL prevalences as high as 53% have been detected [112]; c) German Wildlife Foundation; published online at: http://www.deutschewildtierstiftung.de/de/schuetzen/arten-schuetzen/rothirsch/verbreitungskarte/ (last accessed: 11/28/12); d) Data from 2007, prior to intensive density reduction measures. Study areas were: 1: South Central Spain; 2: Central-East Portugal; 3: Woodchester Park; 4: overall data; 5: North Eastern Lower Peninsula; 6: Riding Mountain National Park and surroundings (elk), Wood buffalo National Park (wood bison); 7: overall data; 8: Kruger National Park, 9: Southern Bavaria; 10: North-Western Italy; 11: North-Western Minnesota. Apparent prevalence was based on the isolation of *M. bovis* and/or *M. caprae*, except: 1 (red deer): TBL prevalence (in areas of known bTB infection); 3: culture and/or serological testing; 4: official sampling (testing method not provided); 6 (wood bison): live animal testing (caudal fold test and/or fluorescent polarisation assay); 7: testing method not provided. Methods for estimating population densities were not provided in most cases, except: 1 (red deer): head-light counts and distance sampling (average density calculated by first author from data provided); 3: capture-mark-recapture; 6 (elk): density calculated by first author from population and map data provided in [107], 9: estimation from hunting bags; 10: driving census; 11: helicopter survey. Prevalence categories: Low: 1–5%; Low-medium: 5–10%; Medium: 10–20%; Medium-high: 20–40%; High: 40–50%. Density categories: Low: 0–5 individuals (ind.)/km^2^; Medium: 5–20 ind./km^2^; High: >20 ind./km^2^.

Part of the risk factors for aggregation and bTB maintenance directly or indirectly result from human influence: Intensive wildlife management (notably at hunting farms or clubs), including practices such as supplementary feeding, watering and fencing [49]–[51], supplementary feeding by non-hunters [42] and decreased genetic variability [52], [53]; and leaving viscera of hunted animals in the fields, which makes potentially infected organs accessible for scavengers (offal consumption) [22], [52], [54], [55]. Further factors favoring aggregation and disease maintenance, like certain social behaviors [24], [56]–[58] and environmental conditions [50], [59]–[61] are largely out of human control. Available data on several maintenance hosts indicate that medium to high bTB prevalences are almost consistently associated to high population densities or a highly gregarious lifestyle (wood bison, African buffalo), in contrast to spillover hosts, which show markedly lower bTB prevalences and lower population densities (Table 3).

#### Telephone survey

Data from our study areas on anthropogenic factors found to influence the risk of bTB maintenance in wildlife (as identified in the literature review) are summarized in Table 4. None of the hunting officials reported the presence of specific aggregation sites of either wild boar or red deer. Supplemental feeding of wild ungulates is prohibited by law in the cantons of Saint Gall and Tessin, as well as in FL. In the cantons of Thurgovia and Grisons, it is not legally regulated for wild boar and red deer but neither recommended nor widely practiced. In the canton of Geneva, wildlife feeding is uncommon and cantonal law provides the possibility to forbid it on a case-by-case basis, should private people engage in such activities. Offal of hunted wild ungulates is regularly left in the fields in almost all study areas. Exceptions are Geneva, where all wild boar viscera are disposed as slaughterhouse waste, and Thurgovia, where roughly half of the hunters have the possibility to eviscerate their animal at slaughterhouse-like facilities and dispose the viscera accordingly. Areas of private property, where wild ungulates are intensively managed and hunted similarly to hunting farms or clubs in Spain or Michigan [49]–[51], do not exist in any of the study areas.

**Table 4:** Assessment of the situation in our study areas regarding risk factors for bTB maintenance.

					Situation of identified risk			
Study area	Species	Prevalence	Density (individuals/km^2^)		factors within study areas			
			Reported	Category	(1)	(2)	(3)	(4)
**Geneva**	Wild boar	0% (95%CI	10.6	Medium	No	No	No	No
		0–5.9%)						
**Thurgovia**	Wild boar	0% (0–11.6)*	No data	-	No	No	(Yes)	No
**Saint Gall**	Red deer	0% (0–7.6)	0.5-2.5	Low	No	No	Yes	No
**Grisons**	Red deer	0% (0–4.3)	9.7	Medium	No	No	Yes	No
**Tessin**	Wild boar	0% (0–4.9)	No data	-	No	No	Yes	No
	Red deer	0% (0–4.1)	No data	-	No	No	Yes	No
**Liechtenstein**	Red deer	0% (0–7.4)	2–4	Low	No	No	Yes	No

* Due to organizational reasons, sampling took place only during the 2010/11 hunting season. Apparent prevalence was calculated based on combined results of PCR investigations on tissue samples and of isolation attempts of *M. bovis* and/or *M. caprae*. Methods for estimating population densities were: capture-resight (Geneva); calculations by the first author using data obtained by direct counts conducted by game wardens in the Swiss National Park, where hunting is prohibited (Grisons; for this canton, no data from the exact location of sampling regions were available); head-light counts (Saint Gall and Liechtenstein). The situation regarding risk factors within the study areas was assessed by a telephone survey among hunting officials. The following questions were asked: (1) Do aggregation sites exist, where wild boar and/or red deer frequently gather in high numbers?; (2) Is supplemental feeding of wild boar and/or red deer permitted and/or commonly practiced?; (3) Is offal of hunted wild boar and red deer regularly left in the fields?; (4) Are there areas of private property where wild boar and red deer are intensively managed and hunted? (similar to the hunting industry abroad, e.g. in Spain).

## Discussion

Our study is the first to assess the situation of bTB, a currently (re)-emerging disease in European wildlife, in the potential maintenance hosts red deer and wild boar in various areas of CH and FL. *M. bovis* and *M. caprae* were not detected by a combined PCR and culture protocol in any of the sampled animals, and the development of a wildlife reservoir is currently unlikely. However, we report for the first time infections with a mycobacterium of the MTBC, *M. microti,* in Swiss wild boar.

### Laboratory analysis

Culture of mycobacteria succeeded in 77 animals, including 13 cases with macroscopic TBL, but the majority of these was negative by PCR_C_ for MTBC and thus classified as atypical mycobacteria. Contamination due to field sampling [62] and infection with mycobacteria of the *M. avium* complex, known to occur in wild boar and common in domestic pigs [37], [63], [64], have to be considered here. The significantly more frequent detection of atypical mycobacteria in wild boar, compared with red deer, may be attributable to the foraging habits of wild boar, which includes digging in the ground; this behavior is more likely to expose wild boar to ubiquitous mycobacteria.

The PCR protocol we used for the detection of MTBC mycobacteria has shown high specificity [44], [65], and appeared more sensitive than culture in our case. Mycobacteria of the MTBC were detected by PCR_T_ in six wild boar, all of which presented TBL, while only three of these animals were also positive by PCR_C_. However, the applied PCR protocol has been validated only for respiratory specimens and false-positive results have been reported from non-respiratory sample material [44], [65]. Nevertheless, sensitivity of PCR_T_ may be limited as only a small tissue portion is used for analysis and both the distribution and total amount of mycobacteria within an infected tissue may vary. Regarding culture, sensitivity may also be affected by various factors [66]. For example, the amount of viable mycobacteria within the affected tissue is highly variable and depends on both the chronicity of the lesion and the quality of the submitted sample [42], [66]–[68]. Also, the use of harsh decontamination protocols, which is necessary due to the long incubation time, further reduces sensitivity [42].

Finally, only part of the required five lymphatic tissues were submitted for examination in 64% of the sampled animals; this may have decreased the sensitivity of our protocol because the presence of mycobacteria may be confined to certain regional lymph nodes only.

Nevertheless, because macroscopically identified TBL are non-specific for bTB, a number of differential diagnoses have to be considered for the observed lesions, such as granulomas of parasitic or fungal origin [69] and infections with *Staphylococcus*, *Streptococcus*, *Actinobacillus* or *Actinomyces* spp. [42]. *Rhodococcus equi* is increasingly detected in slaughter pigs, both with and without macroscopic TBL, and has been found in mandibular lymph nodes of 12.4% of sampled wild boar from Hungary [70]–[73]. Additionally, first cases of *Corynebacterium ulcerans* have recently been reported in two wild boar from Southern Germany [74].

### Occurrence of *M. microt*

Infection with *M. microti* was confirmed by spoligotyping in two out of six PCR_T_ positive animals. Spoligotyping was performed on DNA extracted directly from tissue, as we did not succeed in obtaining pure cultures of MTBC mycobacteria but growth of atypical mycobacteria likely outcompeted MTBC mycobacteria. Strains of *M. microti* can be particularly difficult to culture and slow-growing [75]–[78], compared even to *M. bovis*, and thus overgrowth of *M. microti* with atypical mycobacteria is even more likely. However, our laboratory protocol was selected with regards to the detection of *M. bovis* and *M. caprae*, and other protocols may have been more suitable for the detection of *M. microti* [77], [79].

*M. microti* has been isolated from both wild boar and domestic pigs with TBL before [34], [80], and was recently detected in wild boar presenting macroscopic lesions in Northern Italy (M. Pacciarini, personal communication). It has been detected in many further mammal hosts (e.g. [81]), including diseased humans [78], [82], South American camelids [81], [82], and cats [77].

### Surveillance strategy for bTB in Swiss and Liechtenstein wildlife

Disease surveillance in wildlife in CH and FL consists of national scanning surveillance programs regularly complemented by targeted, risk-based investigations. Between 2006 and 2011 the FIWI staff has performed full necropsies, including histology in most cases, on 520 animals belonging to species known to be potential *M. bovis*/*M. caprae* hosts (badger, red fox *Vulpes vulpes*, roe deer, red deer, wild boar) [28], [83], [84], but bTB suspicion was raised in none of the analyzed animals (FIWI archives, unpublished data). Furthermore, two previous cross-sectional studies on bTB in farmed deer and free-ranging wild boar, respectively, had not detected any infection with *M. bovis* or *M. caprae* [37], [38].

Overall, the combination of data from targeted and scanning surveillance obtained with various diagnostic protocols (necropsy, histology, culture, PCR) does not suggest the occurrence of bTB in wildlife in CH and FL so far. However, as financial restrictions limited sample sizes per study area in the present study, the occurrence of bTB cannot be completely ruled out (see 95% CI, Table 4). Low bTB prevalences have indeed been reported in both wild boar and cervids in other countries, including those where these species are maintenance hosts [34], [42], [59]. Also, in a few of the sampled wild boar MTBC mycobacteria were detected by PCR only and could not be further identified.

Continued disease awareness and good collaboration with field partners constitute prerequisites for long-term effective bTB surveillance, with the restraint that bTB detection in the fields may be impaired by the absence of visible lesions [32], [48], [85]. Considering that the collection of lymphatic tissues from hunted animals is laborious and the current laboratory methods expensive and time-consuming, additional diagnostic tools that are more convenient for large sample sizes, may be valuable for further surveys. Recently, a serological test for the detection of *M. bovis* antibodies was evaluated for wild boar, thus providing an attractive option for future bTB screenings at population scale in this species [86].

However, in a region yet unaffected by bTB, surveillance efforts should not be confined to wildlife alone. During the summer months, Swiss and Liechtenstein livestock from different herds are frequently brought to mountain pastures in neighboring countries including regions with documented bTB occurrence [87]. In the autumn, these animals return to their original farms. Cattle movements in general have proven to be the most important introductory route of bTB into a herd [88]. Furthermore, it is usually spillover from cattle to wild hosts that accounts for the first bTB cases in wildlife, where subsequently a reservoir may or may not develop (e.g. [22], [24], [89], [90]). Contacts between livestock and wild ruminants on Alpine pastures, and between outdoor domestic pigs and wild boar, are regularly observed in Switzerland [91], [92] pointing at existing potential spillover pathways between livestock and wildlife. Therefore, disease awareness is essential also among meat inspectors and veterinarians.

### Risk factors for bTB maintenance worldwide

Among risk factors for bTB maintenance, aggregation in its different forms plays a central role. This reinforces the notion that despite the alleged tenacity of mycobacteria, environmental contamination in general does not play a major role in bTB transmission (e.g., [23], [41], [59], [93], [94]). Moreover, the role of aggregation as a dominant risk factor is reflected in the presence of “hot-spots” of infection in many countries bearing a wildlife reservoir [23], [50], [89], [95], [96]. It has been suggested that such disease hot-spots may constitute “steady state systems”, and even if individuals infect others outside of the hot-spot during dispersal or movement within their home range, these transmission incidents are apparently inefficient to sustain an infection cycle [95].

Data on bTB prevalence and densities of maintenance hosts from the same geographical area were generally difficult to obtain. Furthermore, diagnostic approaches and methods for estimating population densities varied widely (or were not indicated), highlighting the urgent need for harmonized procedures in wildlife health science [97]. However, despite limited comparability, compiled data illustrate the link between bTB prevalence, host density and epidemiological role in wild populations.

### Risk of reservoir emergence in CH and FL

**Figure 6:**
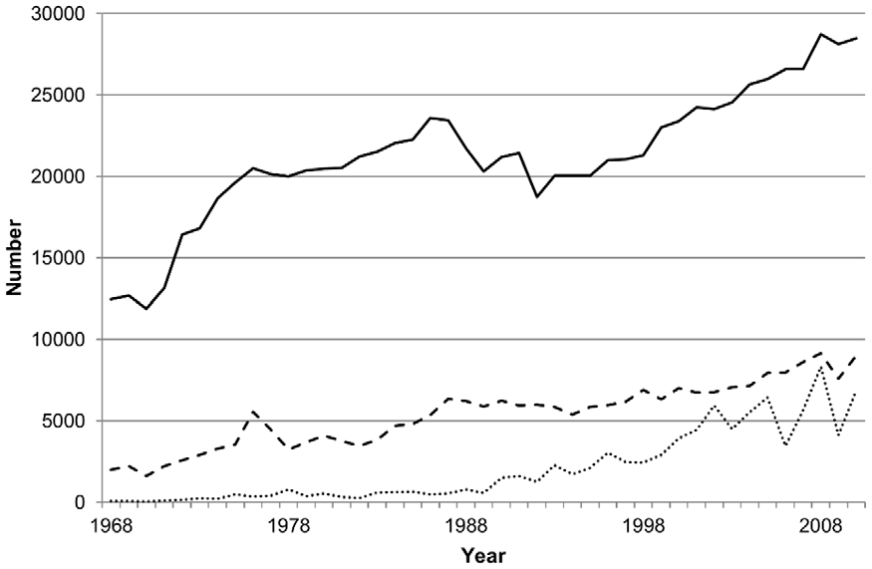
Increase of population numbers and hunting bags of red deer and wild boar in Switzerland. Red deer counts (black line); red deer hunting bag (dashed line); wild boar hunting bag (dotted line). No wild boar counts available. (Source: Swiss hunting statistics: http://www.wild.uzh.ch/jagdst/).

It is not unlikely that the “hot spot concept” mentioned above is applicable to the Alpine situation: Despite (1) the presence of a current infection focus in Austria within a radius of about 50 km from the CH and FL borders [33], (2) the known seasonal migration of red deer between these three countries (ongoing telemetry study; personal communication, A. Duscher), and (3) reported migration distances of red deer of up to 25 km in an Alpine environment [98], bTB does not seem to have crossed these borders so far. Moreover, when considering risk factors associated to the maintenance of bTB in wildlife reservoirs worldwide, the situation in our study areas does not seem favorable for the future development of a wildlife reservoir. In particular, the absence of an intensive wildlife management fostering high population densities, as practiced at private hunting farms or clubs elsewhere, and the lack of widespread feeding of wild ungulates or of further aggregation sites, point towards a comparatively low risk of reservoir emergence in our red deer or wild boar populations at present. Information on wild ungulate densities in our study areas was only available for local study sites and overall population estimates on a cantonal and national level exist only for red deer (Table 1 & Figure 6). However, hunting statistics together with roadkill data, as indicators for population dynamics, show increasing population trends for both study species (Figure 6; [99]). Also, the common practice of leaving offal in the fields presents a potential risk for disease transmission, should bTB be introduced into our wildlife populations at some point. Finally, some discrepancies between official recommendations and field practice may occur: A recent questionnaire survey among game wardens in the canton of Grisons revealed that wildlife feeding is apparently still carried out in some areas, and that red deer visit cattle feeding sites in the winter [91]. Therefore, a more thorough assessment of the situation in the field is warranted.

## Conclusion

The merit of early and adequate response to disease emergence was recently demonstrated by the reaction to an outbreak of bTB in Minnesota, USA, where the establishment of a disease reservoir in a potential maintenance host, the white-tailed deer, was successfully prevented [100]. Choosing a like-minded foresightful approach, we found little indication for the presence of bTB in Swiss and Liechtenstein wildlife today. Yet, in the face of increasing population numbers of wild ungulates and bTB (re)-emergence in European wildlife and livestock, this situation cannot be considered static. Our study should serve as a baseline for future investigations and more importantly as a further incentive to continue a reasonable wildlife management strategy, especially concerning the control of potential aggregation factors, including rising population densities.

## References

[1] Aranaz A, Cousins D, Mateos A, Dominguez L (2003) Elevation of *Mycobacterium tuberculosis* subsp. *caprae* Aranaz et al. 1999 to species rank as *Mycobacterium caprae* comb. nov., sp. nov. Int J Syst Evol Microbiol 53: 1785–1789. 14657105 10.1099/ijs.0.02532-0

[2] Cavanagh R, Begon M, Bennett M, Ergon T, Graham IM, et al (2002) *Mycobacterium microti* infection (vole tuberculosis) in wild rodent populations. J Clin Microbiol 40: 3281–3285. 12202566 10.1128/JCM.40.9.3281-3285.2002 PMC130808

[3] Bouakaze C, Keyser C, Gonzalez A, Sougakoff W, Veziris N, et al (2011) Matrix-assisted laser desorption ionization-time of flight mass spectrometry-based single nucleotide polymorphism genotyping assay using iPLEX gold technology for identification of *Mycobacterium tuberculosis* complex species and lineages. J Clin Microbiol 49: 3292–3299. 21734028 10.1128/JCM.00744-11 PMC3165613

[4] Cousins DV, Bastida R, Cataldi A, Quse V, Redrobe S, et al (2003) Tuberculosis in seals caused by a novel member of the *Mycobacterium tuberculosis* complex: *Mycobacterium pinnipedii* sp nov. Int J Syst Evol Microbiol 53: 1305–1314. 13130011 10.1099/ijs.0.02401-0

[5] Amanfu W (2006) The situation of tuberculosis and tuberculosis control in animals of economic interest. Tuberculosis (Edinb) 86: 330–335. 16644282 10.1016/j.tube.2006.01.007

[6] Briones V, de Juan L, Sanchez C, Vela AI, Galka M, et al (2000) Bovine tuberculosis and the endangered Iberian lynx. Emerg Infect Dis 6: 189–191. 10756155 10.3201/eid0602.000214 PMC2640855

[7] Michel AL, Bengis RG, Keet DF, Hofmeyr M, Klerk LM, et al (2006) Wildlife tuberculosis in South African conservation areas: implications and challenges. Vet Microbiol 112: 91–100. 16343819 10.1016/j.vetmic.2005.11.035

[8] Wobeser G (2009) Bovine tuberculosis in Canadian wildlife: An updated history. Can Vet J 50: 1169–1176. 20119541 PMC2764465

[9] LoBue PA, Enarson DA, Thoen CO (2010) Tuberculosis in humans and animals: an overview. Int J Tuberc Lung Dis 14: 1075–1078. 20819249

[10] European Food Safety Authority (2012) The European Union Summary Report on Trends and Sources of Zoonoses, Zoonotic Agents and Food-borne Outbreaks in 2010. EFSA Journal 2012 10: 442. 10.2903/j.efsa.2018.5500 PMC7009540 32625785

[11] Prodinger WM, Eigentler A, Allerberger F, Schonbauer M, Glawischnig W (2002) Infection of red deer, cattle, and humans with *Mycobacterium bovis* subsp. *caprae* in western Austria. J Clin Microbiol 40: 2270–2272. 12037107 10.1128/JCM.40.6.2270-2272.2002 PMC130709

[12] Rodriguez E, Sanchez LP, Perez S, Herrera L, Jimenez MS, et al (2009) Human tuberculosis due to *Mycobacterium bovis* and *M. caprae* in Spain, 2004–2007. Int J Tuberc Lung Dis 13: 1536–1541. 19919773

[13] Cvetnić Ž, Katalinic-Jankovic V, Sostaric B, Spicic S, Obrovac M, et al (2007) *Mycobacterium caprae* in cattle and humans in Croatia. Int J Tuberc Lung Dis 11: 652–658. 17519097

[14] Schiller I, Oesch B, Vordermeier HM, Palmer MV, Harris BN, et al (2010) Bovine tuberculosis: a review of current and emerging diagnostic techniques in view of their relevance for disease control and eradication. Transbound Emerg Dis 57: 205–220. 20561288 10.1111/j.1865-1682.2010.01148.x

[15] de la Rua-Domenech R, Goodchild AT, Vordermeier HM, Hewinson RG, Christiansen KH, et al (2006) Ante mortem diagnosis of tuberculosis in cattle: A review of the tuberculin tests, gamma-interferon assay and other ancillary diagnostic techniques. Res Vet Sci 81: 190–210. 16513150 10.1016/j.rvsc.2005.11.005

[16] Gortázar C, Delahay RJ, McDonald RA, Boadella M, Wilson GJ, et al.. (2011) The status of tuberculosis in European wild mammals. Mammal Rev: 1–14.

[17] Haydon DT, Cleaveland S, Taylor LH, Laurenson MK (2002) Identifying reservoirs of infection: a conceptual and practical challenge. Emerg Infect Dis 8: 1468–1473. 12498665 10.3201/eid0812.010317 PMC2738515

[18] Ashford RW (1997) What it takes to be a reservoir host. Belg J Zool 127: 85–90.

[19] Ashford RW (2003) When is a reservoir not a reservoir? Emerg Infect Dis 9: 1495–1496. 14725261 10.3201/eid0911.030088 PMC3035539

[20] Corner LAL (2006) The role of wild animal populations in the epidemiology of tuberculosis in domestic animals: How to assess the risk. Vet Microbiol 112: 303–312. 16326039 10.1016/j.vetmic.2005.11.015

[21] Power AG, Mitchell CE (2004) Pathogen spillover in disease epidemics. Am Nat 164 Suppl 5S79–89. 15540144 10.1086/424610

[22] Nugent G (2011) Maintenance, spillover and spillback transmission of bovine tuberculosis in multi-host wildlife complexes: a New Zealand case study. Vet Microbiol 151: 34–42. 21458931 10.1016/j.vetmic.2011.02.023

[23] Morris RS, Pfeiffer DU (1995) Directions and issues in bovine tuberculosis epidemiology and control in New Zealand. N Z Vet J 43: 256–265. 16031864 10.1080/00480169./1995.35904

[24] De Vos V, Bengis RG, Kriek NPJ, Michel A, Keet DF, et al (2001) The epidemiology of tuberculosis in free-ranging African buffalo (*Syncerus caffer*) in the Kruger National Park, South Africa. Onderstepoort J Vet Res 68: 119–130. 11585089

[25] O'Brien DJ, Schmitt SM, Fitzgerald SD, Berry DE (2011) Management of bovine tuberculosis in Michigan wildlife: Current status and near term prospects. Vet Microbiol 151: 179–187. 21414734 10.1016/j.vetmic.2011.02.042

[26] Nishi JS, Shury T, Elkin BT (2006) Wildlife reservoirs for bovine tuberculosis (*Mycobacterium bovis*) in Canada: Strategies for management and research. Vet Microbiol 112: 325–338. 16343817 10.1016/j.vetmic.2005.11.013

[27] Corner LA, Murphy D, Gormley E (2011) *Mycobacterium bovis* infection in the Eurasian badger (*Meles meles*): the disease, pathogenesis, epidemiology and control. J Comp Pathol 144: 1–24. 21131004 10.1016/j.jcpa.2010.10.003

[28] Balseiro A, Rodriguez O, Gonzalez-Quiros P, Merediz I, Sevilla IA, et al (2011) Infection of Eurasian badgers (*Meles meles*) with *Mycobacterium bovis* and *Mycobacterium avium* complex in Spain. Vet J 90: e21–25. 10.1016/j.tvjl.2011.04.012 21612958

[29] Sobrino R, Martín-Hernando MP, Vicente J, Aurtenetxe O, Garrido JM, et al (2008) Bovine tuberculosis in a badger (*Meles meles*) in Spain. Vet Rec 163: 159–160. 18677001 10.1136/vr.163.5.159

[30] Santos N, Correia-Neves M, Ghebremichael S, Kallenius G, Svenson SB, et al (2009) Epidemiology of *Mycobacterium bovis* infection in wild boar (*Sus scrofa*) from Portugal. J Wildl Dis 45: 1048–1061. 19901381 10.7589/0090-3558-45.4.1048

[31] Zanella G, Durand B, Hars J, Moutou F, Garin-Bastuji B, et al (2008) *Mycobacterium bovis* in wildlife in France. J Wildl Dis 44: 99–108. 18263825 10.7589/0090-3558-44.1.99

[32] Gerstmair E (2011) Validierung molekularbiologischer und immunologischer Nachweisverfahren für die Tuberkulose bei Rindern und Tuberkulosemonitoring beim Rotwild. PhD Dissertation, Ludwig Maximilian University of Munich, Germany. 135 p.

[33] Entstrasser-Müller C (2011) Tbc im Oberen Lechtal gemeinsam bekämpfen. Innsbruck: Tiroler Landesregierung. Available: http://www.tirol.gv.at/presse/meldungen/meldung/artikel/tbc-im-oberen-lechtal-gemeinsam-bekaempfen/?no_cache=1&cHash=a75c9d6810. Accessed 11/28/12.

[34] Dondo A, Zoppi S, Rossi F, Chiavacci L, Barbaro A, et al (2007) Mycobacteriosis in wild boar: Results of 2000 -2006 activity in Northern Italy. Épidémiol et Santé Anim 51: 35–42.

[35] Bouvier G, Burgisser H, Schneider PA (1957) Observations sur les maladies du gibier, des oiseaux et des poissons faites en 1955 en 1956. Schweiz Arch Tierheilkd 99: 461–477.

[36] Bouvier G (1963) Transmission possible de la tuberculose et de la brucellose du gibier à l'homme et aux animaux domestiques et sauvages. Bull Off Int Epizooties 59: 433–436.

[37] Leuenberger R (2004) Surveillance of wild boar in Switzerland: Prevalence of infections relevant to domestic pigs. PhD Dissertation, University of Basel, Switzerland. 98 p.

[38] Wyss D, Giacometti M, Nicolet J, Burnens A, Pfyffer GE, et al (2000) Farm and slaughter survey of bovine tuberculosis in captive deer in Switzerland. Vet Rec 147: 713–717. 11140930

[39] Thrusfield M, Ortega C, de Blas I, Noordhuizen JP, Frankena K (2001) WIN EPISCOPE 2.0: improved epidemiological software for veterinary medicine. Vet Rec 148: 567–572. 11370882 10.1136/vr.148.18.567

[40] Martín-Hernando MP, Höfle U, Vicente J, Ruiz-Fons F, Vidal D, et al (2007) Lesions associated with *Mycobacterium tuberculosis* complex infection in the European wild boar. Tuberculosis (Edinb) 87: 360–367. 17395539 10.1016/j.tube.2007.02.003

[41] Zanella G, Duvauchelle A, Hars J, Moutou F, Boschiroli ML, et al (2008) Patterns of lesions of bovine tuberculosis in wild red deer and wild boar. Vet Rec 163: 43–47. 18621995 10.1136/vr.163.2.43

[42] Schmitt SM, Fitzgerald SD, Cooley TM, Bruning-Fann CS, Sullivan L, et al (1997) Bovine tuberculosis in free-ranging white-tailed deer from Michigan. J Wildl Dis 33: 749–758. 9391958 10.7589/0090-3558-33.4.749

[43] Buijtels PCA, Petit PLC (2005) Comparison of NaOH-N-acetyl cysteine and sulfuric acid decontamination methods for recovery of mycobacteria from clinical specimens. J Microbiol Methods 62: 83–88. 15823396 10.1016/j.mimet.2005.01.010

[44] Causse M, Ruiz P, Gutierrez-Aroca JB, Casal M (2011) Comparison of two molecular methods for rapid diagnosis of extrapulmonary tuberculosis. J Clin Microbiol 49: 3065–3067. 21653775 10.1128/JCM.00491-11 PMC3147762

[45] Kamerbeek J, Schouls L, Kolk A, vanAgterveld M, van Soolingen D, et al (1997) Simultaneous detection and strain differentiation of *Mycobacterium tuberculosis* for diagnosis and epidemiology. J Clin Microbiol 35: 907–914. 9157152 10.1128/jcm.35.4.907-914.1997 PMC229700

[46] Cadmus S, Palmer S, Okker M, Dale J, Gover K, et al (2006) Molecular analysis of human and bovine tubercle bacilli from a local setting in Nigeria. J Clin Microbiol 44: 29–34. 16390943 10.1128/JCM.44.1.29-34.2006 PMC1351927

[47] Smith NH, Upton P (2011) Naming spoligotype patterns for the RD9-deleted lineage of the Mycobacterium tuberculosis complex; www.Mbovis.org.Infect Genet Evol. 10.1016/j.meegid.2011.08.002 21855653

[48] Gavier-Widen D, Cooke MM, Gallagher J, Chambers MA, Gortázar C (2009) A review of infection of wildlife hosts with *Mycobacterium bovis* and the diagnostic difficulties of the 'no visible lesion' presentation. N Z Vet J 57: 122–131. 19521460 10.1080/00480169.2009.36891

[49] O'Brien DJ, Schmitt SM, Fitzgerald SD, Berry DE, Hickling GJ (2006) Managing the wildlife reservoir of *Mycobacterium bovis*: The Michigan, USA, experience. Vet Microbiol 112: 313–323. 16376030 10.1016/j.vetmic.2005.11.014

[50] Vicente J, Höfle U, Garrido JM, Fernandez-de-Maria IG, Acevedo P, et al (2007) Risk factors associated with the prevalence of tuberculosis-like lesions in fenced wild boar and red deer in south central Spain. Vet Res 38: 451–464. 17425933 10.1051/vetres:2007002

[51] Castillo L, Fernández-Llario P, Mateos C, Carranza J, Benítez-Medina JM, et al (2010) Management practices and their association with *Mycobacterium tuberculosis* complex prevalence in red deer populations in Southwestern Spain. Prev Vet Med 98: 58–63. 21131079 10.1016/j.prevetmed.2010.11.008

[52] Gortázar C, Vicente J, Boadella M, Ballesteros C, Galindo RC, et al (2011) Progress in the control of bovine tuberculosis in Spanish wildlife. Vet Microbiol 151: 170–178. 21440387 10.1016/j.vetmic.2011.02.041

[53] Acevedo-Whitehouse K, Vicente J, Gortázar C, Höfle U, Fernandez-de-Mera IG, et al (2005) Genetic resistance to bovine tuberculosis in the Iberian wild boar. Mol Ecol 14: 3209–3217. 16101786 10.1111/j.1365-294X.2005.02656.x

[54] Naranjo V, Gortázar C, Vicente J, de la Fuente J (2008) Evidence of the role of European wild boar as a reservoir of *Mycobacterium tuberculosis* complex. Vet Microbiol 127: 1–9. 18023299 10.1016/j.vetmic.2007.10.002

[55] Vieira-Pinto M, Alberto J, Aranha J, Serejo J, Canto A, et al (2011) Combined evaluation of bovine tuberculosis in wild boar (*Sus scrofa*) and red deer (*Cervus elaphus*) from Central-East Portugal. Eur J Wildl Res 57: 1189–1201.

[56] Joly DO, Messier F (2004) Factors affecting apparent prevalence of tuberculosis and brucellosis in wood bison. J Anim Ecol 73: 623–631.

[57] Blanchong JA, Scribner KT, Kravchenko AN, Winterstein SR (2007) TB-infected deer are more closely related than non-infected deer. Biol Lett 3: 103–105. 17443977 10.1098/rsbl.2006.0547 PMC2373800

[58] Vicente J, Delahay RJ, Walker NJ, Cheeseman CL (2007) Social organization and movement influence the incidence of bovine tuberculosis in an undisturbed high-density badger *Meles meles* population. J Anim Ecol 76: 348–360. 17302842 10.1111/j.1365-2656.2006.01199.x

[59] Lees VW (2004) Learning from outbreaks of bovine tuberculosis near Riding Mountain National Park: applications to a foreign animal disease outbreak. Can Vet J 45: 28–34. 14992251 PMC539224

[60] Ayele WY, Neill SD, Zinsstag J, Weiss MG, Pavlik I (2004) Bovine tuberculosis: an old disease but a new threat to Africa. Int J Tuberc Lung Dis 8: 924–937. 15305473

[61] Miller R, Kaneene JB, Fitzgerald SD, Schmitt SM (2003) Evaluation of the influence of supplemental feeding of white-tailed deer (*Odocoileus virginianus*) on the prevalence of bovine tuberculosis in the Michigan wild deer population. J Wildl Dis 39: 84–95. 12685071 10.7589/0090-3558-39.1.84

[62] Norton JH, Duffield BJ, Coward AJ, Hielscher RW, Nicholls RF (1984) A necropsy technique for cattle to eliminate contamination of lymph-nodes by mycobacteria. Aust Vet J 61: 75–76. 6743146 10.1111/j.1751-0813.1984.tb15521.x

[63] Cvetnić Ž, Špičić S, Benić M, Katalinić-Janković V, Pate M, et al (2007) Mycobacterial infection of pigs in Croatia. Acta Vet Hung 55: 1–9. 17385551 10.1556/AVet.55.2007.1.1

[64] Komijn RE, de Haas PE, Schneider MM, Eger T, Nieuwenhuijs JH, et al (1999) Prevalence of *Mycobacterium avium* in slaughter pigs in the Netherlands and comparison of IS1245 restriction fragment length polymorphism patterns of porcine and human isolates. J Clin Microbiol 37: 1254–1259. 10203466 10.1128/jcm.37.5.1254-1259.1999 PMC84743

[65] Kim JH, Kim YJ, Ki CS, Kim JY, Lee NY (2011) Evaluation of Cobas TaqMan MTB PCR for detection of M*ycobacterium tuberculosis* . J Clin Microbiol 49: 173–176. 21048015 10.1128/JCM.00694-10 PMC3020466

[66] Cassidy JP (2006) The pathogenesis and pathology of bovine tuberculosis with insights from studies of tuberculosis in humans and laboratory animal models. Vet Microbiol 112: 151–161. 16310979 10.1016/j.vetmic.2005.11.031

[67] Bollo E, Ferroglio E, Dini V, Mignone W, Biolatti B, et al (2000) Detection of *Mycobacterium tuberculosis* complex in lymph nodes of wild boar (*Sus scrofa*) by a target-amplified test system. J Vet Med Ser B 47: 337–342. 10.1046/j.1439-0450.2000.00354.x 10900824

[68] Griffin JF, Buchan GS (1994) Aetiology, pathogenesis and diagnosis of *Mycobacterium bovis* in deer. Vet Microbiol 40: 193–205. 8073625 10.1016/0378-1135(94)90055-8

[69] Rohonczy EB, Balachandran AV, Dukes TW, Payeur JB, Rhyan JC, et al (1996) A comparison of gross pathology, histopathology, and mycobacterial culture for the diagnosis of tuberculosis in elk (*Cervus elaphus*). Can J Vet Res 60: 108–114. 8785715 PMC1263815

[70] Shitaye JE, Parmova I, Matlova L, Dvorska L, Horvathova A, et al (2006) Mycobacterial and *Rhodococcus equi* infections in pigs in the Czech Republic between the years 1996 and 2004: the causal factors and distribution of infections in the tissues. Vet Med (Praha) 51: 497–511.

[71] Komijn RE, Wisselink HJ, Rijsman VM, Stockhofe-Zurwieden N, Bakker D, et al (2007) Granulomatous lesions in lymph nodes of slaughter pigs bacteriologically negative for *Mycobacterium avium* subsp. *avium* and positive for *Rhodococcus equi* . Vet Microbiol 120: 352–357. 17126501 10.1016/j.vetmic.2006.10.031

[72] Makrai L, Kobayashi A, Matsuoka M, Sasaki Y, Kakuda T, et al (2008) Isolation and characterisation of *Rhodococcus equi* from submaxillary lymph nodes of wild boars (*Sus scrofa*). Vet Microbiol 131: 318–323. 18499361 10.1016/j.vetmic.2008.04.009

[73] Makrai L, Takayarna S, Denes B, Hajtos I, Sasaki Y, et al (2005) Characterization of virulence plasmids and serotyping of *Rhodococcus equi* isolates from submaxillary lymph nodes of pigs in Hungary. J Clin Microbiol 43: 1246–1250. 15750091 10.1128/JCM.43.3.1246-1250.2005 PMC1081261

[74] Contzen M, Sting R, Blazey B, Rau J (2011) *Corynebacterium ulcerans* from diseased wild boars. Zoonoses Public Health 58: 479–488. 21824349 10.1111/j.1863-2378.2011.01396.x

[75] Oevermann A, Pfyffer GE, Zanolari P, Meylan M, Robert N (2004) Generalized tuberculosis in llamas (*Lama glama*) due to *Mycobacterium microti* . J Clin Microbiol 42: 1818–1821. 15071059 10.1128/JCM.42.4.1818-1821.2004 PMC387549

[76] Zanolari P, Robert N, Lyashchenko KP, Pfyffer GE, Greenwald R, et al (2009) Tuberculosis caused by *Mycobacterium microti* in South American camelids. J Vet Intern Med 23: 1266–1272. 19709353 10.1111/j.1939-1676.2009.0377.x

[77] Smith NH, Crawshaw T, Parry J, Birtles RJ (2009) *Mycobacterium microti*: More diverse than previously thought. J Clin Microbiol 47: 2551–2559. 19535520 10.1128/JCM.00638-09 PMC2725668

[78] van Soolingen D, van der Zanden AGM, de Haas PEW, Noordhoek GT, Kiers A, et al (1998) Diagnosis of *Mycobacterium microti* infections among humans by using novel genetic markers. J Clin Microbiol 36: 1840–1845. 9650922 10.1128/jcm.36.7.1840-1845.1998 PMC104938

[79] de Jong E, Rentenaar RJ, van Pelt R, de Lange W, Schreurs W, et al (2009) Two cases of *Mycobacterium microti*-induced culture-negative tuberculosis. J Clin Microbiol 47: 3038–3040. 19571033 10.1128/JCM.00772-09 PMC2738074

[80] Taylor C, Jahans K, Palmer S, Okker M, Brown J, et al (2006) *Mycobacterium microti* isolated from two pigs. Vet Rec 159: 59–60. 10.1136/vr.159.2.59-a 16829603

[81] Kremer K, van Soolingen D, van Embden J, Hughes S, Inwald J, et al (1998) *Mycobacterium microti*: more widespread than previously thought. J Clin Microbiol 36: 2793–2794. 9742014 10.1128/jcm.36.9.2793-2794.1998 PMC105214

[82] Frank W, Reisinger EC, Brandt-Hamerla W, Schwede I, Handrick W (2009) *Mycobacterium microti* - pulmonary tuberculosis in an immunocompetent patient. Wien Klin Wochenschr 121: 282–286. 19562286 10.1007/s00508-009-1164-0

[83] Balseiro A, Oleaga A, Orusa R, Robetto S, Zoppi S, et al (2009) Tuberculosis in roe deer from Spain and Italy. Vet Rec 164: 468–470. 19363229 10.1136/vr.164.15.468

[84] Martin-Atance P, Palomares F, Gonzalez-Candela M, Revilla E, Cubero MJ, et al (2005) Bovine tuberculosis in a free ranging red fox (*Vulpes vulpes*) from Doñana National Park (Spain). J Wildl Dis 41: 435–436. 16107680 10.7589/0090-3558-41.2.435

[85] O'Brien DJ, Schmitt SM, Berry DE, Fitzgerald SD, Lyon TJ, et al (2008) Estimating the true prevalence of *Mycobacterium bovis* in free-ranging elk in Michigan. J Wildl Dis 44: 802–810. 18957636 10.7589/0090-3558-44.4.802

[86] Boadella M, Lyashchenko K, Greenwald R, Esfandiari J, Jaroso R, et al (2011) Serologic tests for detecting antibodies against *Mycobacterium bovis* and *Mycobacterium avium* subspecies *paratuberculosis* in Eurasian wild boar (*Sus scrofa scrofa*). J Vet Diagn Invest 23: 77–83. 21217031 10.1177/104063871102300111

[87] Schiller I, RayWaters W, Vordermeier HM, Jemmi T, Welsh M, et al (2011) Bovine tuberculosis in Europe from the perspective of an officially tuberculosis free country: Trade, surveillance and diagnostics. Vet Microbiol 151: 153–159. 21439740 10.1016/j.vetmic.2011.02.039

[88] Johnston WT, Gettinby G, Cox DR, Donnelly CA, Bourne J, et al (2005) Herd-level risk factors associated with tuberculosis breakdowns among cattle herds in England before the 2001 foot-and-mouth disease epidemic. Biol Lett 1: 53–56. 17148126 10.1098/rsbl.2004.0249 PMC1629052

[89] Shury TK, Bergeson D (2011) Lesion distribution and epidemiology of *Mycobacterium bovis* in elk and white-tailed deer in south-western Manitoba, Canada. Vet Med Int 2011: 591980. 21776351 10.4061/2011/591980 PMC3135165

[90] Gallagher J, Clifton-Hadley RS (2000) Tuberculosis in badgers; a review of the disease and its significance for other animals. Res Vet Sci 69: 203–217. 11124091 10.1053/rvsc.2000.0422

[91] Casaubon J, Vogt H-R, Stalder H, Hug C, Ryser-Degiorgis M-P (2012) Bovine viral diarrhea virus in free-ranging wild ruminants in Switzerland: low prevalence of infection despite regular interactions with domestic livestock. BMC Vet Res 8: 204. 23107231 10.1186/1746-6148-8-204 PMC3514304

[92] Wu N, Abril C, Thomann A, Grosclaude E, Doherr MG, et al (2012) Risk factors for contacts between wild boar and outdoor pigs in Switzerland and investigations on potential *Brucella suis* spill-over. BMC Vet Res 8: 116. 22817843 10.1186/1746-6148-8-116 PMC3464720

[93] Michel AL, de Klerk LM, Gey van Pittius NC, Warren RM, van Helden PD (2007) Bovine tuberculosis in African buffaloes: observations regarding *Mycobacterium bovis* shedding into water and exposure to environmental mycobacteria. BMC Vet Res 3: 23. 17900356 10.1186/1746-6148-3-23 PMC2151946

[94] Witmer G, Fine AE, Gionfriddo J, Pipes M, Shively K, et al (2010) Epizootiologic survey of *Mycobacterium bovis* in wildlife and farm environments in northern Michigan. J Wildl Dis 46: 368–378. 20688630 10.7589/0090-3558-46.2.368

[95] Hickling GJ (2002) Dynamics of bovine tuberculosis in wild white-tailed deer in Michigan. Michigan Bovine Tuberculosis Bibliography and Database Paper 27: 1–34.

[96] Cheeseman CL, Jones GW, Gallagher J, Mallinson PJ (1981) The population structure, density and prevalence of tuberculosis (*Mycobacterium bovis*) in badgers (*Meles meles*) from four areas in south-west England J Appl Ecol. 18: 795–804.

[97] Kuiken T, Ryser-Degiorgis M-P, Gavier-Widen D, Gortázar C (2011) Establishing a European network for wildlife health surveillance. Rev Sci Tech 30: 755–761. 22435188 10.20506/rst.30.3.2067

[98] Georgii B, Schroder W (1983) Home range and activity patterns of male red deer (*Cervus elaphus L*) in the Alps. Oecologia 58: 238–248. 28310585 10.1007/BF00399224

[99] Wu N, Abril C, Hinic V, Brodard I, Thur B, et al (2011) Free-ranging wild boar: a disease threat to domestic pigs in Switzerland? J Wildl Dis 47: 868–879. 22102657 10.7589/0090-3558-47.4.868

[100] Carstensen M, Doncarlos MW (2011) Preventing the establishment of a wildlife disease reservoir: a case study of bovine tuberculosis in wild deer in Minnesota, USA. Vet Med Int 2011: 413240. 21647335 10.4061/2011/413240 PMC3103846

[101] Acevedo P, Vicente J, Höfle U, Cassinello J, Ruiz-Fons F, et al (2007) Estimation of European wild boar relative abundance and aggregation: a novel method in epidemiological risk assessment. Epidemiol Infect 135: 519–527. 16893488 10.1017/S0950268806007059 PMC2870594

[102] Vicente J, Höfle U, Fernandez-De-Mera IG, Gortázar C (2007) The importance of parasite life history and host density in predicting the impact of infections in red deer. Oecologia 152: 655–664. 17401583 10.1007/s00442-007-0690-6

[103] Clifton-Hadley RS, Wilesmith JW, Richards MS, Upton P, Johnston S (1995) The occurrence of *Mycobacterium bovis* infection in cattle in and around an area subject to extensive badger (*Meles meles*) control. Epidemiol Infect 114: 179–193. 7867737 10.1017/s0950268800052031 PMC2271337

[104] Rogers LM, Cheeseman CL, Mallinson PJ, Clifton-Hadley R (1997) The demography of a high-density badger (*Meles meles*) population in the west of England. J Zool (Lond) 242: 705–728.

[105] Delahay RJ, Langton S, Smith GC, Clifton-Hadley RS, Cheeseman CL (2000) The spatio-temporal distribution of *Mycobacterium bovis* (bovine tuberculosis) infection in a high-density badger population. J Anim Ecol 69: 428–441.

[106] European Food Safety Authority (2011) The European Union Summary Report on Trends and Sources of Zoonoses, Zoonotic Agents and Food-borne Outbreaks in 2009. EFSA Journal 2011 9: 378.

[107] Lees VW, Copeland S, Rousseau P (2003) Bovine tuberculosis in elk (*Cervus elaphus manitobensis*) near Riding Mountain National Park, Manitoba, from 1992 to 2002. Can Vet J 44: 830–831. 14601681 PMC340302

[108] Rodwell TC, Kriek NP, Bengis RG, Whyte IJ, Viljoen PC, et al (2001) Prevalence of bovine tuberculosis in African buffalo at Kruger National Park. J Wildl Dis 37: 258–264. 11310876 10.7589/0090-3558-37.2.258

[109] Marsan A, Spano S (1995) Management attempts of wild boar (*Sus scrofa L.*): First results and outstanding researches in Northern Apennines (Italy). J Mt Ecol 3: 219–221.

[110] Dini V, Ferroglio E, Serraino A, Mignone W, Sanguinetti V, et al (2004) Epidemiologia delle Microbatteriosi del cinghiale in Liguria. J Mt Ecol 7: 145–153.

[111] Sleeman DP, Davenport J, More SJ, Clegg TA, Collins JD, et al (2009) How many Eurasian badgers *Meles meles L.* are there in the Republic of Ireland? Eur J Wildl Res 55: 333–344.

[112] Coleman JD, Jackson R, Cooke MM, Grueber L (1994) Prevalence and spatial distribution of bovine tuberculosis in brushtail possums on a forest-scrub margin. N Z Vet J 42: 128–132. 16031762 10.1080/00480169.1994.35802

